# Whole Genome Sequencing Analysis of African Orthobunyavirus Isolates Reveals Naturally Interspecies Segments Recombinations between Bunyamwera and Ngari Viruses

**DOI:** 10.3390/v15020550

**Published:** 2023-02-16

**Authors:** Moufid Mhamadi, Idrissa Dieng, Anna S. Dolgova, Cheikh Talibouya Touré, Mignane Ndiaye, Moussa Moïse Diagne, Babacar Faye, Anna S. Gladkikh, Vladimir G. Dedkov, Amadou Alpha Sall, Ousmane Faye, Oumar Faye

**Affiliations:** 1 Virology Department, Institut Pasteur de Dakar, Dakar 12900, Senegal; 2 Parasitology Department, Université Cheikh Anta Diop de Dakar, Dakar 10700, Senegal; 3 Saint Petersburg Pasteur Institute, 197101 Saint Petersburg, Russia

**Keywords:** Bunyamwera, serogroup, segmented RNA, genetic recombination

## Abstract

Bunyamwera virus is the prototype of the Bunyamwera serogroup, which belongs to the order *Bunyavirales* of the *Orthobunyavirus* genus in the Peribunyaviridae family. Bunyamwera is a negative-sense RNA virus composed of three segments S, M, and L. Genetic recombination is possible between members of this order as it is already documented. Additionally, it can lead to pathogenic or host range improvement, if it occurs with viruses of public health and agricultural importance such as Rift Valley fever virus and Crimea–Congo hemorrhagic fever virus. Here, we characterize five African *Orthobunyavirus* viruses from different geographical regions. Our results suggest that the five newly characterized strains are identified as Bunyamwera virus strains. Furthermore, two of the five strains sequenced in this study are recombinant strains, as fragments of their segments are carried by Ngari and Bunyamwera strains. Further investigations are needed to understand the functional impact of these recombinations.

## 1. Introduction

The Bunyavirales are one of the largest viral orders, containing more than 350 viruses including several viruses of public health and agricultural importance such as Rift Valley fever virus (RVF) and Crimean–Congo hemorrhagic fever virus [1]. As Bunyavirales members, *Orthobunyaviruses* include a heterogeneous viral group, which Bunyamwera serogroup is one of the largest. The Bunyamwera serogroup regroups arthropod-borne virus members, Bunyamwera virus (BUNV), Ngari virus (NRIV), and Batai virus (BATV), are some of the most isolated members of this serogroup in Africa [2].

BUNV, BATV, and NRIV have a tri-segmented RNA genome: small (S), medium (M), and large (L). The S segment encodes the nucleocapsid protein (N) and a non-structural protein (NSs), which are translated from overlapping open reading frames (ORF). The M encodes glycoproteins (Gn and Gc) and a non-structural protein (NSm), and the L mRNA encodes the RNA-dependent RNA polymerase (RDRP) [3].

Bunyamwera virus (BUNV) was first isolated in Uganda in 1943. Since that, BUNV was isolated from many arthropods such as *Aedes* mosquitoes and *Amblyomma* and *Hyalomma* ticks [4,5]. BUNV is widespread in several African countries, North America, and South America. To note, the virus was reported in Uganda, Tanzania, Mozambique, Nigeria, Guinea, South Africa, the Democratic Republic of Congo, Senegal, Guinea, the Ivory Coast, Nigeria, Cameroon, the Central African Republic, Kenya, Uganda, South Africa, Madagascar, Argentina, and the United States of America [4,6,7]. BUNV causes disease in both humans and domestic animals. The disease associated with BUNV has been reported to cause mild symptoms, such as fever, joint pain, and rash, in many mammals, including humans [4]. In 2015, BUNV infection was associated with encephalitis/neurological disease and abortion in three horses in Argentina [6]. 

Firstly, isolated from *Anopheles maculipennis* in Czechoslovakia in 1950, the Batai virus (BATV) is nowadays the Bunyamwera serogroup member with the largest geographical distribution. BATV was isolated in Asia (India and Malaysia), Europe (Ukraine, Croatia, Norway, Montenegro, Slovakia, Belarus, the Czech Republic, Serbia, Bosnia, Italy, Hungary, Romania, Austria, Portugal, Germany, Finland, and Sweden), and Africa (Uganda) [2]. In livestock animals, BATV can cause severe symptoms including inborn deficiency and abortion [8], and in humans, flu-like symptoms are frequently associated with BATV infection [9]. 

Genetic reassortment and genetic recombination are the driving forces in the evolution of viruses from the Bunyamwera serogroup [10,11]. Recombination events had already been demonstrated between the snowshoe hare virus and La Crosse virus in co-infected cell culture and also in co-infected arthropod hosts [11]. Additionally, natural reassortment had been documented between four orthobunyavirus members: between Bunyamwera virus and Batai virus, and between Potosi virus and Main Drain virus [12,13,14,15,16]. For instance, Ngari (NRIV) possesses the M segment of the Batai virus (BATV) combined with the S and L segments from BUNV [15]. The current demarcation criteria for interspecific differentiation established for bunyaviruses, BUNV, and NRIV belong to *Bunyamwera orthobunyavirus* spp. [16]. They are two different genetic variants of the same species—*Bunyamwera orthobunyavirus* sp. [16]. As the Batai virus is known to cause more severe symptoms than the Bunyamwera virus, the reassortment event likely affected the emergence of NRIV, making it more virulent and capable of inducing severe and fatal hemorrhagic fever with a similarity of its clinical manifestations with RVF in humans and small ruminants [2,17,18]. For the record, NRIV is frequently isolated during Rift Valley fever virus (RVF) outbreaks, which could be due to the similarity of their symptoms or signs [14,19].

A recent study shows that this segment reassortment between Batai and Bunyamwera probably occurred in co-infected mammalian cells [20] and that NRIV is only reported in Africa (Senegal, Burkina Faso, Mauritania, the Central African Republic, Madagascar, Kenya, and Somalia) [14,15,16,17,18,19,20,21]. These data combined with the fact that many of the known *orthobunyavirus* members use the same hosts and are located in the same area showed enormous potential for the emergence of reassortment and recombinant bunyaviruses with pathogenic potential improvement or host range [18].

In Senegal, few are known about Orthobunyavirus, so a genetic characterization of *orthobunyavirus* isolates from the Institut Pasteur de Dakar (IPD) biobank using Next Generation Sequencing (NGS) was aimed in this study.

## 2. Materials and Methods

### 2.1. Viral Stock Preparation

The viral strains used in this study were provided by the WHO collaborating center for reference and research for arboviruses and hemorrhagic fever viruses (WHOCC) in the Virology Department of the Institut Pasteur de Dakar (IPD). Those strains are described in Table 1. The viral stock was prepared by inoculating 200 µL of each viral strain into Vero cells monolayer continuous cell lines (ATCC, USA) in Leibovitz 15 (L-15) growth medium (GibcoBRL, Grand Island, NY, USA) supplemented with 5% fetal bovine serum (FBS) (GibcoBRL, Grand Island, NY, USA), 1% penicillin-streptomycin, and 0.05% fungizone (Sigma, Germany). After the observation of the cytopathic effect, supernatants were harvested and stored at −80 °C until further use.

### 2.2. Conventional RT-PCR

RNA was extracted from supernatants using the QIAamp RNA Viral Kit (Qiagen GmbH, Heiden, Germany) following the manufacturer’s recommendations. Using the RNA extracts, we tested the presence of the *Orthobunyavirus* S segment by RT-PCR using TITAN one-tube RT-PCR kit (Boehringer Mannheim Biochemicals) reagents and the primers previously described [22]. For the reaction mixture, we added 4 μL of RNA with the mixture reaction composed of 10.5 μL H_2_O, 5 μL of buffer, 2.5 μL of each primer, and 0.5 μL of the enzyme. The PCR was performed following the manufacturer’s recommendations. PCR products were visualized on 2% ethidium bromide-stained agarose gel.

### 2.3. Next-Generation Sequencing 

RNA extract was used for Illumina sequencing. Briefly, the host’s background RNA was depleted as previously described using mammal ribosomal RNA-specific primers and RNAseH enzyme (NEB). Purified viral RNA was reverse transcribed into first-stranded cDNA using the Invitrogen SuperScript IV Reverse Transcriptase kit (Thermo Fisher, Waltham, MA, USA). Then, amplicons were tagged using the “index tags” incorporated into the Nextera kit XT DNA Library Preparation kit (Illumina, San Diego, CA, USA). The obtained libraries were quantified, normalized, pooled, and loaded onto an Illumina MiSeq platform with 151 bp paired-end reads using the Miseq reagents kit v3, according to the manufacturer’s protocol (Illumina, San Diego, CA, USA). Demultiplexing, removal of sequencing adapters, and genome mapping were performed by a homemade package incorporated into the IPD server. Genetic identity was determined by a nucleotide Basic Local Alignment Search Tool (nBLAST).

### 2.4. Sequence Analysis

The MUSCLE alignment algorithm was used for multiple nucleotides sequences alignments in VIPR software [23] using nucleotide sequences classified as Bunyamwera, Batai, Kairi, and Ngari [16] (Table A1). Recombination was tested with Recombination Detection Program (RDP) version 4 (RDP4), a software package suitable for statistical identification and characterization of recombination events in nucleotide sequences [24]. For this purpose, the 3 segments were linearized and aligned. Then, the seven primary exploratory recombination signal detection methods provided by the package (the original RDP method, GENECONV, BOOTSCAN/RECSCAN, MAXCHI, Chimaera, 3SEQ, and SISCAN) were used for the evaluation of recombination events using defaults parameters. Analysis by pairwise comparison of L-segment’s full-length amino acid was performed to check if our strains belonged to the Bunyamwera group according to the current ICTV demarcation criteria [25]. Furthermore, the genetic distance between population was calculated to measure the genetic divergence between groups of sequences, and fixation index (FST) values were estimated to estimate the genetic differentiation of the tested viruses using MEGA [25] and DNAsp [26] software, respectively. Genetic distance and FST values vary between 0 and 1. That means that populations with a value of 0 are genetically similar whereas a value of 1 indicates that these populations are genetically divergent. Maximum likelihood (ML) tree constructions were performed using IQ-TREE software [27]. The appropriate model for each segment was also calculated and the robustness of the tree topology was tested during 1000 non-parametric bootstrap analyses. Kairi virus was used as an outgroup. For the record, genetic recombination is an evolutionary process that results in an exchange of genetic information between segments. Recombination takes place between viruses of the same group or subgroup, whereas, reassortment takes place between viruses of different groups. Reassortment is a non-classical mechanism of recombination that does not involve breaking the nucleic acid of the viral genome.

## 3. Results

To fulfill the gap of the lack of data about the genetic characterization of the Bunyamwera viruses isolated in Senegal, we aim to genetically characterize Orthobunyavirus strains that are available in the WHO collaborating center for arboviruses and viral hemorrhagic fever (WHOCC) located in IPD. 

After sequencing, five complete genomes were obtained and nucleotide BLAST showed that the segments of the ArB218, ArN31, ArMsam263, ArY380, and ArY52 strains were identified as Bunyamwera viruses with high nucleotide similarities (>95%). Except for the strain ArB218 which showed 98.12% of nucleotide identity for the segment S of NRIV strain 9800535 (JX857328.1) for the S segment. An analysis of the genetic distances shows that our isolates are closer to Bunyamwera than Ngari and Batai (Table 2).

The fixation indices (FST) measured between populations also show that these newly sequenced strains are closer to the Bunyamwera virus (Table 3).

Analyses of the amino acid similarities of the L segments between our strains and the sampled NRIV, BATV, and BUNV sequences reveal 98.82% of similarities with BUNV (Table 4).

**Figure 1:**
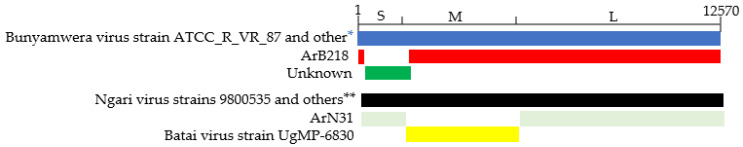
Representation of recombination events between our strains and the sampled sequences. *: Bunyamwera virus (reference strain). **: Ngari virus strains (15Guidimaka_ME, 428Trarza_ME, 9800521, D28542_4e, HKV66, HKV141, and Adrar).

The recombination test identified two recombinants among our five strains and the breakpoint was detected by some of the seven primary exploratory recombination signal detection methods provided by the package. Recombination analysis shows that the S and L of the Ngari virus strains (15Guidimaka_ME, 428Trarza_ME, 9800521, D28542_4e, HKV66, HKV141, and Adrar) result from recombination with the S and L segments of the newly characterized strain ArN31, with a recombination signal detected by all the seven detections methods (*p*-value < 0.001) (Figure 1). Moreover, six methods (RDP, BOOTSCAN/RECSCAN, MAXCHI, Chimaera, 3SEQ, and SISCAN) indicate that the segments S and L from the Bunyamwera virus strain ATCC_R_VR_87 and Bunyamwera virus (reference strain) carried fragments from S, M, and L of the ArB218 strains (*p*-value < 0.001) (Figure 1).

**Figure 2:**
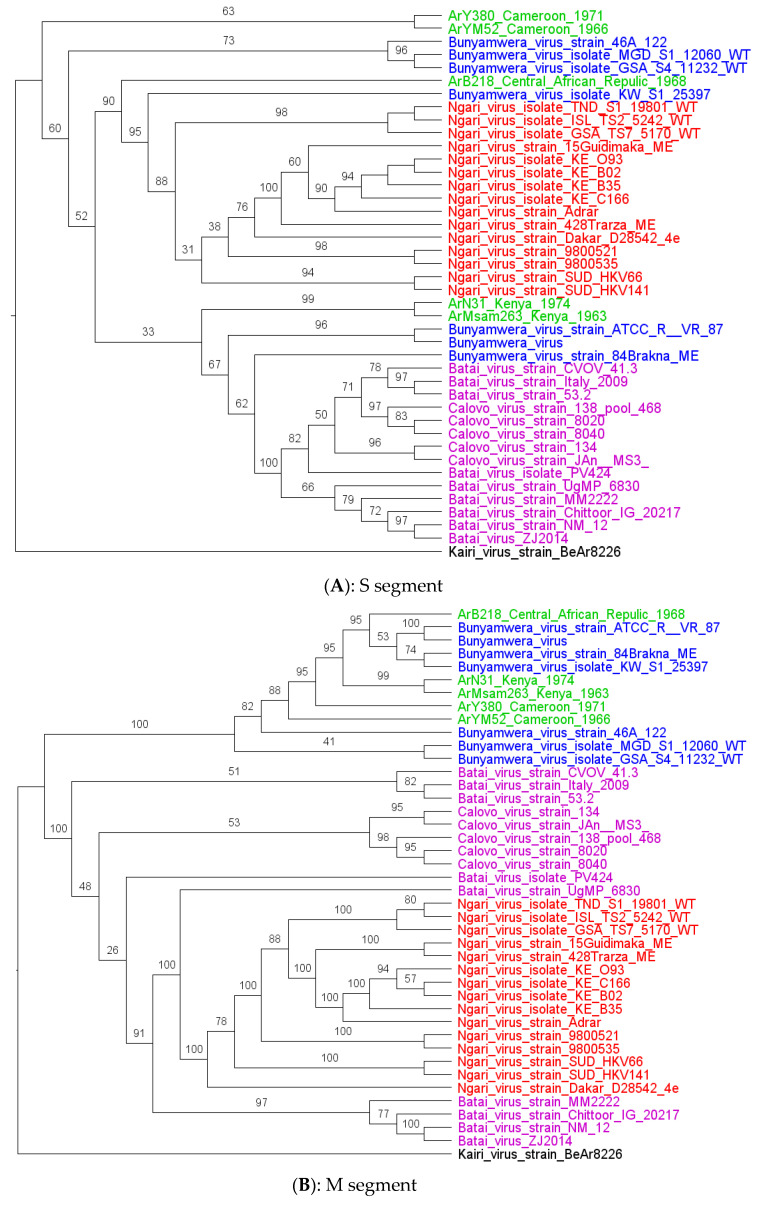

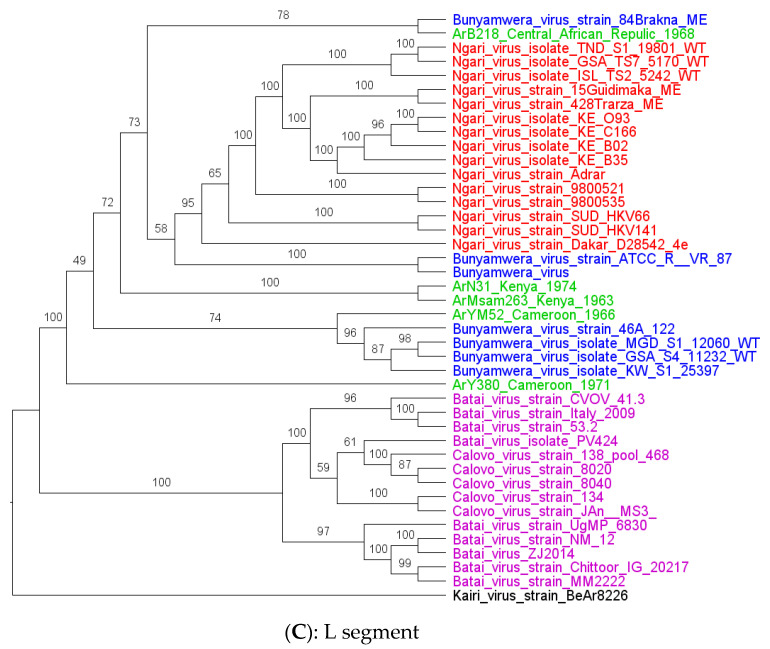
Maximum likelihood trees were obtained from the S (**A**), M (**B**), and L (**C**) segments analysis. Legends: study strain (green), Batai virus (pink), Bunyamwera (red), and Ngari (blue). The strain characterized in this study is colored purple.

Phylogenetic analysis shows that all the strains characterized in this study had clustered with Bunyamwera virus strains for all segments, except for the S and L segments of ArB218, which had clustered in the base of the node of the S and L segments of the NRIV strains group (Figure 2A–C) with the two recombinants Bunyamwera virus strains, KW_S1_25397 and 84 Brakna ME, respectively, for the S and L segments. More particularly, the phylogenetic analyses of the four other strains of this study show that they are grouped by two by country (Ar Ms am 263_Kenya with Ar Na 31_Kenya and Ar YM 52_Cameroon with Ar Y 380/69_Cameroon) for the S and M segments. For the L segment, Ar Ms am 263 and Ar Na 31 remain grouped, whereas Ar YM 52 and Ar Y 380/69 were no longer grouped by two for the L segments. These strains from Cameroon seem to be close to some Bunyamwera strains from Kenya.

## 4. Discussion

In this study, we have genetically characterized five Bunyamwera strains provided by the WHOCC in IPD. The results of BLAST show that our five orthobunyavirus strains were identified as belonging to the Bunyamwera serogroup as all their three segments are identified as a Bunyamwera virus (BUNV) except for the S Segment of ArB 218, which was identified as an Ngari virus (NRIV). This result is strengthened by an analysis of the genetic distance, amino acid similarities of the L segments, and the differentiation index (FST), which indicate that these newly sequenced strains are the Bunyamwera virus. Otherwise, two recombination events were detected during our analysis. Ngari strains (15Guidimaka_ME, 428Trarza_ME, Ngari virus strain 9800535, 9800521, D28542_4e, HKV66, HKV141, and Adrar) result from recombination with S and L segments of our strain ArN31. Additionally, Bunyamwera virus strain ATCC_R_VR_87 results from recombination with S, M, and L segments of the ArB218 strain. Natural genetic segments exchange between Bunyamwera serogroup members has already occurred. Genomic data showed that NRIV carried the M segment of BATV and the L and S segments of BUNV [15,18]. According to the current knowledge, BUNV and NRIV share the same geographic areas and host (arthropod and vertebrate) [2]. Then, this segment recombination between our strains ArN31 and ArB218 with Ngari strain SUD-HKV141, Ngari virus strain 9800535, and Bunyamwera virus strain ATCC_R_VR_87 could have occurred in co-infected vectors or in the infected vertebrate hosts [12,28]. This result was strengthened by a phylogenetic analysis where the S and the L segment of ArB 218 clustered on the basal node with S and L segments of the NRIV strains group when the M segment clustered with M of BUNV groups. As the S segment encodes for the NSs, which is known to block the production of type I IFN, it blocks transcription and translation, induces apoptosis, and inhibits apoptosis. This S-segment recombination could lead to the emergence of a new virus with a greater or lesser potential to constitute a threat to humans and livestock, as already documented with NRIV [15,29].

Furthermore, among the strains (Ar Ms am 263, Ar Na 31, Ar YM 52, and Ar Y 380/69) that fall in the Bunyamwera genetic cluster for all the three segments, the newly sequenced Ar Ms am 263 and Ar Na 31 from Kenya were grouped for the S, M, and L segments in a BUNV cluster, even if they were isolated 10 years apart. This could be explained by the fact that they were isolated from hosts that belong to the same genus. Besides the strains from Cameroon, Ar YM 52 and Ar Y 380/69 did not group by two except for the S segments. Additionally, the L segment is close to some of the Bunyamwera isolates from Kenya GSA/S4/11232_WT, MGD/S1/12060_WT, and 46A-122.

Finally, the genetic analyses of five newly characterized strains revealed that these strains are characterized by the Bunyamwera virus and that two of them are recombined with the Ngari and Bunyamwera strains. Further investigations are needed to elucidate the impact of these recombinations on the biology of this virus, such as growth kinetics, pathogenesis analysis, and vector competence, which are needed to understand the role of this genetic recombination.

## Figures and Tables

**Table 1:** Strains used in this study.

Strains ID	Year	Host	Place	Passages
Ar YM 52	1966	*Aedes* sp.	Cameroon	9
Ar Y 380/69	1971	*Aedimorphus capensis*	Cameroon	5
Ar Ms am 263	1963	*Mansonioides uniformis*	Kenya	6
Ar Na 31	1974	*Mansonioides Africa*	Kenya	6
ArB 218	1968	*Culex* sp.	Central African Republic	4

**Table 2:** Estimates of net genetic distance between groups.

	Batai	Study Strains	Bunyamwera	Ngari
Batai		0.01263	0.01244	0.00951
Study strains	0.47021		0.00091	0.00822
Bunyamwera	0.46175	0.00843		0.00787
Ngari	0.31337	0.21865	0.21064	

The number of amino acid substitutions per site from the estimation of the net average between groups of sequences is shown. Standard error estimate(s) are shown (colored in blue) above the diagonal and were obtained by a bootstrap procedure (1000 replicates).

**Table 3:** Estimates of FST between groups.

	Batai	Study Strains	Bunyamwera	Ngari
Batai				
Study strains	0.79341			
Bunyamwera	0.77318	0.10286		
Ngari	0.74199	0.83386	0.79616	

The estimated fixation indices between groups are shown.

**Table 4:** Estimates of L segments amino acid similarities between groups.

	Batai	Study Strains	Bunyamwera	Ngari
Batai		0.0203	0.0205	0.0209
Study strains	0.6400		0.0014	0.0051
Bunyamwera	0.6357	0.0118		0.0047
Ngari	0.6647	0.0623	0.0589	

The number of amino acid substitutions per site from the estimation of the net average between groups of sequences is shown. Standard error estimate(s) are shown (colored in blue) above the diagonal and were obtained by a bootstrap procedure (1000 replicates).

## Data Availability

Not applicable.

## Appendix A

**Table A1:** Viruses used in this study.

Virus Name	Virus Strains
Batai	Batai virus strain Chittoor/IG-20217
	Batai virus strain CVOV 41.3
	Batai virus strain Italy-2009
	Batai virus strain MM2222
	Batai virus strain NM/12
	Batai virus isolate PV424
	Batai virus strain UgMP-6830
	Batai virus ZJ2014 Batai_virus_strain_53.2
	Calovo virus strain 134
	Calovo virus strain 138-pool 468
	Calovo virus strain 8020
	Calovo virus strain 8040
	Calovo virus strain Jan (MS3)
Kairi	Kairi virus strain BeAr8226
Bunyamwera	Bunyamwera virus strain 46A-122
	Bunyamwera virus Bunyamwera virus isolate MGD_S1_12060_WT Bunyamwera virus strain ATCC(R) VR-87 Bunyamwera virus isolate GSA_S4_11232_WT Bunyamwera virus strain 84Brakna ME Bunyamwera_virus_isolate_KW_S1_25397
Newly characterized strains	ArB218_Central African Repulic_1968 ArN31_Kenya_1974 ArMsam263_Kenya_1963 ArY380_Cameroon_1971 ArY52_Cameroon_1966
Ngari	Ngari virus strain 9800521 Ngari virus strain 9800535 Ngari virus strain Adrar Ngari virus strain Dakar D28542/4e Ngari virus strain SUD-HKV66 Ngari virus strain SUD-HKV141 Ngari_virus_isolate_KE_B35 Ngari_virus_isolate_KE_B02 Ngari_virus_isolate_KE_O93 Ngari_virus_isolate_KE_C166 Ngari virus strain 15Guidimaka ME Ngari virus strain 428Trarza ME Ngari virus strain 428Trarza ME Ngari_virus_isolate_ISL_TS2_5242_WT Ngari_virus_isolate_GSA_TS7_5170_WT Ngari_virus_isolate_TND_S1_19801_WT
